# Prevalence and patterns of gender-based violence across adolescent girls and young women in Mombasa, Kenya

**DOI:** 10.1186/s12905-020-01081-8

**Published:** 2020-10-12

**Authors:** Parinita Bhattacharjee, Huiting Ma, Helgar Musyoki, Eve Cheuk, Shajy Isac, Margaret Njiraini, Peter Gichangi, Sharmistha Mishra, Marissa Becker, Michael Pickles

**Affiliations:** 1 Institute for Global Public Health, University of Manitoba, Winnipeg, Canada; 2 Partners for Health and Development in Africa, Nairobi, Kenya; 3 MAP Centre for Urban Health Solutions, Li Ka Shing Knowledge Institute, Toronto, Canada; 4 Department of Medicine, St. Michael’s Hospital, University of Toronto, Toronto, Canada; 5 National AIDS and STI Control Programme, Ministry of Health, Nairobi, Kenya; 6 International Centre for Reproductive Health- Kenya, Mombasa, Kenya; 7 Institute of Medical Sciences, University of Toronto, Toronto, Canada; 8 Institute of Health Policy Management and Evaluation, Dalla Lana School of Public Health, University of Toronto, Toronto, Canada; 9 Department of Infectious Disease Epidemiology, School of Public Health, Imperial College London, London, UK

**Keywords:** Adolescent, Female, Violence, Gender-based violence, Sexual behaviour, Kenya

## Abstract

**Background:**

We sought to estimate the prevalence and describe heterogeneity in experiences of gender-based violence (GBV) across subgroups of adolescent girls and young women (AGYW).

**Methods:**

We used data from a cross-sectional bio-behavioural survey among 1299 AGYW aged 14–24 in Mombasa, Kenya in 2015. Respondents were recruited from hotspots associated with sex work, and self-selected into one of three subgroups: young women engaged in casual sex (YCS), young women engaged in transactional sex (YTS), and young women engaged in sex work (YSW). We compared overall and across subgroups: prevalence of lifetime and recent (within previous year) self-reported experience of physical, sexual, and police violence; patterns and perpetrators of first and most recent episode of physical and sexual violence; and factors associated with physical and sexual violence.

**Results:**

The prevalences of lifetime and recent physical violence were 18.0 and 10.7% respectively. Lifetime and recent sexual violence respectively were reported by 20.5 and 9.8% of respondents. Prevalence of lifetime and recent experience of police violence were 34.7 and 25.8% respectively. All forms of violence were most frequently reported by YSW, followed by YTS and then YCS. 62%/81% of respondents reported having sex during the first episode of physical/sexual violence, and 48%/62% of those sex acts at first episode of physical/sexual violence were condomless. In the most recent episode of violence when sex took place levels of condom use remained low at 53–61%. The main perpetrators of violence were intimate partners for YCS, and both intimate partners and regular non-client partners for YTS. For YSW, first-time and regular paying clients were the main perpetrators of physical and sexual violence. Alcohol use, ever being pregnant and regular source of income were associated with physical and sexual violence though it differed by subgroup and type of violence.

**Conclusions:**

AGYW in these settings experience high vulnerability to physical, sexual and police violence. However, AGYW are not a homogeneous group, and there are heterogeneities in prevalence and predictors of violence between subgroups of AGYW that need to be understood to design effective programmes to address violence.

## Background

There is global recognition that gender-based violence (GBV) significantly impacts public health and human rights [1]. Violence against women takes many forms – physical, sexual, economic, and psychological [2]. Unequal gender power dynamics in relationships, men’s control over women including decision-making, rigid gender roles and low negotiation skills among girls and women, and inequitable gender and social norms are all associated with violence [3]. Community-level tolerance of violence against women and girls facilitates perpetration of GBV [4, 5]. These norms and practices are further reinforced by national or sub-national laws and policies, including how laws that are meant to protect girls and women are implemented [6].

The World Health Organisation (WHO) estimates that 30% of women worldwide have experienced physical and/or sexual intimate partner violence, and 7% have experienced non-partner sexual violence, in their lifetime. Among ever-partnered young women aged 15–24, prevalence of intimate partner violence is 29%. Prevalence of combined intimate partner and non-partner violence ranges from 27% in the WHO European Region to 46% in the African Region [1]. The 2010 Kenya Violence against Children Study (VACS) showed that violence against young women and children is a serious problem in Kenya: 32 and 66% of females aged 18 to 24 reported at least one experience of sexual or physical violence respectively prior to age 18, and 11 and 49% of females aged 13 to 17 reported experiencing some type of sexual violence or physical violence respectively in the past 12 months [7]. Perpetrators of sexual violence included boyfriends and romantic partners, as well as friends/classmates, strangers and family members.

Consequences of exposure to violence have long term effects on girls and women, including increased risk of contraceptive non-use, unwanted pregnancies, unsafe abortions, sexually transmitted infections and low birth weight babies, increased risk of alcohol and substance abuse, as well as self-harm and further victimization in later life [1, 8, 9]. Previous research from Kenya suggests that girls who are victims of violence may experience feelings of hopelessness, anxiety and depression, as well as suicidal thoughts, which may also expose them to further negative health outcomes [7, 8, 10]. Less than 8% of female respondents in VACS who reported sexual violence in the survey had subsequently received any professional help [7].

Violence against women and girls is increasingly visible on the global health and development agenda. However, there are data gaps, and in particular the prevalence, types, and perpetrators of violence against adolescent girls and young women (AGYW) are not well understood. AGYW are also not a homogeneous group. Those engaged in higher risk activities, such as transactional sex or sex work, are more vulnerable than others, and experience different forms of violence, often from different types of perpetrators. Researchers in Mombasa, Kenya, found that women who initiated sex work before 17 years of age were more likely to experience recent physical and sexual violence and verbal abuse from paying partners compared to those who entered sex work after age 17 [11]. Several studies reveal that rape and physical violence against girls and women who engage in transactional sex is common [12, 13]. Conditions of a first sexual encounter, such as a girl’s or woman’s age, use of condoms, and consent during sex, can be indicators of future risk of HIV infection and gender-based violence [14]. However, young women who sell sex or engage in transactional sex remain under-represented both in research on sex workers and on AGYW more generally, as these studies rarely disaggregate outcomes by age or sex work due to ethical or legal concerns [15]. It is important to understand the heterogeneities in vulnerability and experiences of violence to tailor programmes for the specific needs of the subgroups within AGYW.

We sought to address some of the evidence gaps by: (1) estimating the prevalence of partner and non-partner physical and sexual violence among AGYW practicing casual sex, transactional sex and sex work (defined in the methods section) in Mombasa, Kenya; (2) examine the patterns and perpetrators of physical and sexual violence within each subgroup at different time-points; and (3) examine the factors associated with lifetime and recent experience of partner and non-partner physical and sexual violence in the three subgroups.

## Methods

### Study setting and population

We used data from a 2015 cross-sectional, bio-behavioural survey conducted in Mombasa County (population of 1,106,444 including 134,885 adolescent girls and young women aged 14–24 years [16]).

Participants were identified and recruited from sites associated with sex work (“hotspots”), such as bars, guesthouses, nightclubs and public places. These hotspots had been systematically identified using a geographical mapping approach described previously [17]. Participants were selected using a multi-stage cluster sampling approach with probability proportional to the size of the enumerated AGYW population in the hotspots [14]. Peer educators, former or current female sex workers (FSW) from Mombasa, visited selected hotspots and pre-screened individuals who were congregating at the hotspot for eligibility until the pre-allocated sample size for that hotspot was achieved. Interested potential participants were given an invitation card to come to an interview site. Women were invited to participate, and screened for eligibility at a separate confidential study site near the recruitment site. Individuals were eligible to participate if they were cis-gender female age 14–24 years on their last birthday who reported having engaged in vaginal or anal sex at least once in their lifetime. At the interview site, trained research assistants screened and, following written informed consent, enrolled the participants. Of the 1304 women who met the eligibility criteria, 1299 consented to the survey.

Following informed written consent, trained interviewers administered a face-to-face interview in the local language using a structured questionnaire that had been pilot-tested with AGYW including sex workers, and which included questions about past and current experience of sexual and physical violence perpetrated by sex partners, and about police violence. If the participant consented to take part in the biological component of the study, she was introduced to a clinical officer or nurse counsellor who performed HIV rapid testing with pre-and post-test counselling, and collected additional biological samples. All the respondents were subsequently referred to the research partner NGO working with adolescent girls and young sex workers in Mombasa for more HIV and reproductive health services.

The Transitions study received ethical approval from the Kenyatta National Hospital/University of Nairobi Ethics and Research Committee; the Research Permit Committee of the National Commission for Science, Technology and Innovation, Kenya; and the Human Research Ethics Board at the University of Manitoba, Canada. No further permissions were required to access the data used in this study.

### Definitions

In this study, the respondents were told about the three categories used in this study, with clear definitions: young sex workers (YSW), young women engaged in transactional sex, and young women (YTS) engaged in casual sex (YCS). They then self-selected into these categories, based on the definition that they identified with most. The study defined casual sex as when a woman and a man engaged sexually but neither party expects to receive money, gifts or other resources in return; transactional sex as when a woman engages with a man sexually, with the expectation that she would receive money, gifts or other resources in return but the price of sex was not negotiated beforehand [18, 19]; and sex work as transaction between a man and a woman that exchanges money (or gifts or other resources) for sex, where the price of sex is negotiated and both parties have to agree on the price of sex before the sex event is to take place. During the course of the interview participants were asked a series of questions about their sexual partners, their experiences of receiving money, gifts or resources from sexual partners, and about behaviours engaged in during their sexual activity. Based on their self-selected group, each participant was administered a questionnaire that was relevant to their subgroup.

Hotspots were defined as physical locations where people sought sexual partners or had sex. In this study, the hotspots were categorized as either venue-based or non-venue-based. Example typologies of venue-based hotspots included: a) bar/nightclub/casino/hotel (i.e. venues with bars and rooms); b) bar/restaurant/café (i.e. venues with bars but without rooms); c) guesthouse/lodge (i.e. venues with rooms but without bars); d) sex den/brothel; e) local brew den (street kiosks selling mnazi, a palm wine made from naturally fermented coconut tree sap) [20]. Non-venue-based hotspots included a) streets; b) public places such as parks.

### Variables

Respondents were asked about physical and sexual violence in separate questions. A participant was recorded as ‘ever experiencing physical violence’ if she reported that she had ever been physically hurt by a sex partner; and as ‘ever experiencing sexual violence’ if she reported that a man forced her to have sex with him when she was not willing. For each type of violence, missing answers were excluded from subsequent analysis. Respondents were then asked about when the most recent act of each type of violence occurred, and we used this to generate variables for recent experiences of physical and sexual violence, defined as occurring in the previous year. The questionnaire also included items related to the first and most recent experiences of physical and sexual violence, including listing the perpetrator of the violence on each occasion. We also asked about whether anal and/or vaginal sexual intercourse occurred during those episodes of physical and sexual violence, and, if so, whether a condom had been used. Individuals who experienced only one episode of violence were only asked about their first episode of violence. We defined police violence as being physically assaulted or arrested by law enforcement during sex work.

### Analyses

We used descriptive statistics to report the socio-demographic characteristics of the overall and subgroup study populations (YSW, YTS and YCS). We used chi-squared tests to compare proportions. We estimated and compared the prevalence of lifetime (‘ever”) and recent (“previous one year”) experience of physical and sexual violence across subgroups. We then described and compared the pattern (characteristics of first versus most recent experiences of physical and sexual violence) by subgroup. We used chi-squared tests, and Fisher’s exact test when the numbers in each cell were small, to identify factors associated with lifetime and last 1 year experience of physical and sexual violence, stratified by group. Finally, for each violence outcome (lifetime and last 1 year experience of physical and sexual violence respectively), we performed multivariable analyses separately for each subgroup. In each subgroup, and for each violence outcome, we identified the factors associated with that outcome in univariable regression with *p* <  0.1 (results not shown), and used backwards stepwise regression to construct the models, starting with all variables initially identified in univariable regression, and at each step retaining the model with the lowest AIC. We used R (version 3.4.4) to perform analyses.

## Results

### Participants characteristics

Of the 1299 participants, 714 (55.0%) self-classified as young women engaged in casual sex (YCS), 177 (13.6%) as young women engaged in transactional sex (YTS), and 408 (31.4%) as young women engaged in sex work (YSW) (Table 1). Participants were mostly recruited from venue-based hotspots (82.3%), with a slightly lower proportion among the YTS group. The median age of the respondents was 19 years (range 14–24 years), and most (97.2%) could read and write. 20.5% of respondents identified as a current student, but this varied significantly between the subgroups (28.4% YCS; 16.9% YTS; and 8.1% of YSW in school; *p* <  0.001). 12.6% reported any regular source of income (11.2% YCS; 9.6% YTS; and 16.5% YSW; *p* = 0.02) and 38.0% of respondents had ever been pregnant (26.5% YCS; 39.5% YTS; and 57.4% YSW; *p* < 0.001). One in 10 respondents consumed alcohol on a daily basis (0.3% YCS, 6.2% YTS; and 29.7% YSW; *p* < 0.001).

**Table 1:** Descriptive statistics showing socio-demographic characteristics of the study population

Characteristics	Overall, N (%) ***N*** = 1299	YCS N (%) ***N*** = 714	YTS N (%) ***N*** = 177	YSW N (%) ***N*** = 408	***P*** value
Median age, years (IQR)	19 (17–21)	19 (17–21)	19 (18–21)	20 (18–22)	
Can read and write	1262 (97.2%)	693 (97.1%)	172 (97.2%)	397 (97.3%)	0.97
Ever married	62 (4.8%)	25 (3.5%)	10 (5.7%)	27 (6.6%)	0.05
Currently a student	266 (20.5%)	203 (28.4%)	30 (16.9%)	33 (8.1%)	< 0.001
Regular source of income	164 (12.6%)	80 (11.2%)	17 (9.6%)	67 (16.5%)	0.02
Consumed alcohol almost every day or every day in last month	134 (10.3%)	2 (0.3%)	11 (6.2%)	121 (29.7%)	< 0.001
Ever pregnant	493 (38.0%)	189 (26.5%)	70 (39.5%)	234 (57.4%)	< 0.001
Recruited from venue-based hotspot type	1069 (82.3%)	585 (81.9%)	136 (76.8%)	348 (85.3%)	0.04

### Prevalence of gender-based violence

Table 2 gives the lifetime and recent prevalences of physical, sexual and police violence overall and by subgroup. The overall prevalences of lifetime and recent physical violence were 18.0 and 10.7% respectively; both types of violence significantly different between subgroups (each *p* < 0.001), and were most common among YSW, followed by YTS, and finally YCS.

**Table 2:** Prevalence of gender-based violence, overall and by group. *P*-values from chi-squared test

Type of violence	Overall, N (%)	YCS, N (%)	YTS, N (%)	YSW, N (%)	***P***-value
**Physical Violence**					
Ever experienced physical violence	234 (18.0%)	73 (10.2%)	39 (22.0%)	122 (29.9%)	< 0.001
Experienced physical violence in last year	139 (10.7%)	39 (5.5%)	21 (11.9%)	79 (19.4%)	< 0.001
**Sexual Violence**					
Ever experienced sexual violence	266 (20.5%)	97 (13.6%)	50 (28.2%)	119 (29.2%)	< 0.001
Experienced sexual violence in last year	127 (9.8%)	44 (6.2%)	25 (14.1%)	58 (14.2%)	< 0.001
**Police Violence (arrest and or assault)**					
Ever experienced police violence	203 (34.7%)	NA^a^	20 (11.3%)	183 (44.9%)	< 0.001
Experienced police violence last 1 year	151 (25.8%)	NA^a^	12 (6.8%)	139 (34.1%)	< 0.001

^a^The question on police violence was not asked to respondents in YCS subgroup

The prevalences of lifetime and recent sexual violence were 20.5 and 9.8% respectively. In contrast to physical violence, the prevalences of lifetime and recent sexual violence were similar among YSW (29.2 and 14.2%) and YTS (28.2 and 14.1%). The prevalences of lifetime and recent sexual violence were both lowest among YCS (13.6 and 6.2%). The difference between subgroups was significant (*p* < 0.001) for both outcomes.

Among YTS and YSW, experience of police violence was also highly prevalent: 34.7 and 25.8% reported ever or recently experiencing police violence respectively. The prevalence of lifetime and recent police violence was significantly higher among YSW than YTS (*p* < 0.001 for both outcomes).

### Patterns and perpetrators of physical and sexual violence

#### Patterns of physical violence (Table 3)

The pattern of sex during an episode of physical violence, among those reporting physical violence, was similar between the first and most recent episode, with just over 60% having vaginal or anal sex during the episode of physical violence. At both time-points, sexual intercourse during an episode of physical violence varied significantly between subgroups (*p* < 0.001), being most commonly reported by YSW, followed by YTS and YCS. A condom was used in 52.1% of the sex events that occurred during the first episode of physical violence and increased slightly to 60.9% with sex acts that occurred in the most recent episode of physical violence. At both time-points, condom use was higher among YSW (59.6 and 66.7%), followed by YTS (52.2 and 50.0%), and finally YCS (27.6 and 33.3%). The difference between subgroup was significant for first act of physical violence (*p* = 0.01) but not the last act, due to the smaller number reporting.

**Table 3:** Patterns of physical violence

**First Physical Violence**	**Overall, N (%)** ***N*** **= 234**	**YCS, N (%)** ***N*** **= 73**	**YTS, N (%)** ***N*** **= 39**	**YSW, N (%)** ***N*** **= 122**	***P*-value**
Sex (vaginal or anal) during the first episode of physical violence	146 (62.4%)	29 (39.7%)	23 (59.0%)	94 (77.0%)	< 0.001
Used condom in that sexual act	76 (52.1%)	8 (27.6%)	12 (52.2%)	56 (59.6%)	0.01
**Last Physical Violence**	**Overall, N (%)** ***N*** **= 74**	**YCS, N (%)** ***N*** **= 18**	**YTS, N (%)** ***N*** **= 12**	**YSW, N (%)** ***N*** **= 44**	***P*-value**
Sex (vaginal or anal) during the last episode of violence	46 (62.2%)	6 (33.3%)	4 (33.3%)	36 (81.8%)	< 0.001
Used condom in the sexual act	28 (60.9%)	2 (33.3%)	2 (50.0%)	24 (66.7%)	0.32

*P*-values from Fisher’s exact test

### Perpetrators of physical violence (Table 4)

The main perpetrators for the first episode of physical violence are shown in Table 4 and varied by group. Among YCS it was reported as intimate partners (husbands/boyfriends/spouse) (95.9%); for YTS intimate partners (37.8%) and regular non-client partners (37.8%) were most frequent; and for YSW first-time paying clients (37.7%) and regular paying clients (36.9%) were the main perpetrators. While YCS were only asked whether the perpetrator was an intimate partner or other, YTS and YSW were also asked about first-time and regular non-client partners, and YSW were additionally asked about regular and first-time clients. In the most recent episode, there was an increase in regular non-client partner perpetrators for YTS (60.0%) with a corresponding decline in first-time non-client partner perpetrators, while for YSW the main perpetrators remained similar to the first episode.

**Table 4:** Perpetrators of physical violence

**First Physical Violence**	**Overall, N (%)** ***N*** **= 234**	**YCS, N (%)** ***N*** **= 73**	**YTS, N (%)** ***N*** **= 39**	**YSW, N (%)** ***N*** **= 122**
Perpetrator				
- Husband/Spouse/Boyfriends	107 (46.1%)	70 (95.9%)	14 (37.8%)	23 (18.9%)
- Regular paying clients	45 (19.4%)	NA^a^	NA^a^	45 (36.9%)
- First-time paying clients	46 (19.8%)	NA^a^	NA^a^	46 (37.7%)
- Regular non-client partner	19 (8.2%)	NA^a^	14 (37.8%)	5 (4.1%)
- First-time non-client partner	10 (4.3%)	NA^a^	8 (21.6%)	2 (1.6%)
- Others	5 (2.2%)	3 (4.1%)	1 (2.7%)	1 (0.8%)
**Last Physical Violence**	**Overall, N (%)** ***N*** **= 66**	**YCS, N (%)** ***N*** **= 16**	**YTS, N (%)** ***N*** **= 10**	**YSW, N (%)** ***N*** **= 40**
Perpetrator				
- Husband/Spouse/Boyfriends	22 (33.3%)	16 (100.0%)	3 (30.0%)	3 (7.5%)
- Regular paying clients	16 (24.2%)	NA^a^	NA^a^	16 (40.0%)
- First-time paying clients	14 (21.2%)	NA^a^	NA^a^	14 (35.0%)
- Regular non-client partner	12 (18.2%)	NA^a^	6 (60.0%)	6 (15.0%)
- First-time non-client partner	2 (3.0%)	NA^a^	1 (10.0%)	1 (2.5%)
- Others	0 (0.0%)	0 (0.0%)	0 (0.0%)	0 (0.0%)

^a^This question was not asked to the specific subgroup

### Patterns of sexual violence (Table 5)

There was also variation in the pattern of sex between the first and most recent episode of sexual violence. The first episode of sexual violence was slightly more likely to be associated with vaginal or anal sex compared to most recent episode of violence (81.2% versus 69.9%). At both time-points, vaginal or anal sexual intercourse during an episode of sexual violence was most commonly reported by YSW (84.0 and 77.6%) followed by YTS (78.0 and 63.2%) and YCS (79.4 and 53.3%). The difference between the subgroups was not significant during either episode of sexual violence. A condom was used in 37.5% of the sex events that occurred during the first episode of sexual violence and increased to 53.4% in the most recent episode of sexual violence. Condom use among all subgroups was low during the first episode, but during the most recent episode there was a significant difference between subgroups in condom use as condom use during sex at most recent sexual violence was lower than the first sexual violence among YTS and YCS subgroups, but increased among YSW. As a comparison of condom use among these groups in the absence of violence, during the last month 72.2, 69.5 and 68.3% of vaginal/anal sex acts were protected by condoms among all YCS, YTS and YSW respectively.

**Table 5:** Patterns of sexual violence

**First Sexual Violence**	**Overall, N (%)** ***N*** **= 266**	**YCS N (%)** ***N*** **= 97**	**YTS N (%)** ***N*** **= 50**	**YSW N (%)** ***N*** **= 119**	***P*-value**
Sex (vaginal or anal) during that first episode of sexual violence	216 (81.2%)	77 (79.4%)	39 (78.0%)	100 (84.0%)	0.52
Used condom in that sexual act	81 (37.5%)	26 (33.8%)	17 (43.6%)	38 (38.0%)	0.56
**Most Recent Sexual Violence**	**Overall, N (%)** ***N*** **= 83**	**YCS N (%)** ***N*** **= 15**	**YTS N (%)** ***N*** **= 19**	**YSW N (%)** ***N*** **= 49**	***P*-value**
Sex (vaginal or anal) during that most recent episode of violence	58 (69.9%)	8 (53.3%)	12 (63.2%)	38 (77.6%)	0.15
Used condom in that sexual act	31 (53.4%)	2 (25.0%)	4 (33.3%)	25 (65.8%)	0.03

*P*-values from Fisher’s exact test

### Perpetrators of sexual violence (Table 6)

The main perpetrators for the first episode of sexual violence were intimate partners for YCS (65.6%) and YTS (46.9%); and intimate partners (31.9%) and first-time paying clients (23.3%) for YSW. All three subgroups reported many other perpetrators during their first experience of sexual violence including friends, relatives and strangers. For the last episode of sexual violence, the main perpetrators remained the same for YCS, although YTS respondents reported a higher proportion of regular non-client partner perpetrators (46.7%) and a decrease in first-time non-client partner perpetrators compared to the first episode. For YSW the most common perpetrator changed from husband/spouse/boyfriend at first episode to regular paying clients for the most recent episode (47.6%), while almost a quarter of perpetrators were first-time paying client (23.8%). Other perpetrators at last episode were strangers and friends.

**Table 6:** Perpetrators of sexual violence

**First Sexual Violence**	**Overall, N (%)** ***N*** **= 266**	**YCS N (%)** ***N*** **= 97**	**YTS N (%)** ***N*** **= 50**	**YSW N (%)** ***N*** **= 119**
Perpetrator				
- Husband/Spouse/Boyfriends	121 (46.9%)	61 (65.6%)	23 (46.9%)	37 (31.9%)
- Regular paying clients	21 (8.1%)	0 (0.0%)	0 (0.0%)	21 (18.1%)
- First-time paying clients	27 (10.5%)	0 (0.0%)	0 (0.0%)	27 (23.3%)
- Regular non-client partner	16 (6.2%)	0 (0.0%)	10 (20.4%)	6 (5.2%)
- First-time non-client partner	19 (7.4%)	0 (0.0%)	12 (24.5%)	7 (6.0%)
- Others	54 (20.9%)	32 (34.4%)	4 (8.2%)	18 (15.5%)
**Most Recent Sexual Violence**	**Overall, N (%)** ***N*** **= 69**	**YCS N (%)** ***N*** **= 12**	**YTS N (%)** ***N*** **= 15**	**YSW N (%)** ***N*** **= 42**
Perpetrator				
- Husband/Spouse/Boyfriends	21 (30.4%)	9 (75.0%)	6 (40.0%)	6 (14.3%)
- Regular paying clients	20 (29.0%)	0 (0.0%)	0 (0.0%)	20 (47.6%)
- First-time paying clients	10 (14.5%)	0 (0.0%)	0 (0.0%)	10 (23.8%)
- Regular non-client partner	10 (14.5%)	0 (0.0%)	7 (46.7%)	3 (7.1%)
- First-time non-client partner	4 (5.8%)	0 (0.0%)	2 (13.3%)	2 (4.8%)
- Others	4 (5.8%)	3 (25.0%)	0 (0.0%)	1 (2.4%)

### Factors associated with physical and sexual violence

Table 7 and Supplement Table 1 respectively describe the correlates of lifetime and last one-year experience of physical and sexual violence by subgroup. Supplement Table 2 shows the results of multivariable regression analyses for lifetime and last one-year experience of physical and sexual violence by subgroup.

**Table 7:** Potential correlates of lifetime experience of physical and sexual violence by subgroup

**Ever experienced physical violence**	**YCS**			**YTS**			**YSW**		
	**Overall, N (%)** ***N*** **= 714**	**Experienced violence, N (%)** ***N*** **= 73**	***P*** **value**	**Overall, N (%)** ***N*** **= 177**	**Experienced violence, N (%)** ***N*** **= 39**	***P*** **value**	**Overall, N (%)** ***N*** **= 408**	**Experienced violence N (%)** ***N*** **= 122**	***P*** **value**
Aged < 18 years	223 (31.2%)	21 (28.8%)	0.716	40 (22.6%)	12 (30.8%)	0.189	63 (15.4%)	23 (18.9%)	0.212
Can read and write	693 (97.1%)	70 (95.9%)	0.737	172 (97.2%)	38 (97.4%)	1.0	397 (97.3%)	121 (99.2%)	0.227
Regular source of income	80 (11.2%)	7 (9.6%)	0.845	17 (9.6%)	4 (10.3%)	1.0	67 (16.4%)	13 (10.7%)	**0.042**
Consumed alcohol almost every day or every day in last month	2 (0.3%)	2 (2.7%)	**0.010**	11 (6.2%)	7 (17.9%)	**0.003**	121 (29.7%)	50 (41.0%)	**0.001**
Ever pregnant	189 (26.5%)	30 (41.1%)	**0.005**	70 (39.5%)	18 (46.2%)	0.359	234 (57.4%)	84 (68.9%)	**0.002**
Currently student	203 (28.4%)	15 (20.5%)	0.132	30 (16.9%)	3 (7.7%)	0.094	33 (8.1%)	12 (9.8%)	0.429
Venue based hotspot	585 (81.9%)	58 (79.5%)	0.525	136 (76.8%)	35 (89.7%)	**0.032**	348 (85.3%)	103 (84.4%)	0.761
**Ever experienced sexual violence**	**YCS**			**YTS**			**YSW**		
	**Overall, N (%)** ***N*** **= 714**	**Experienced violence, N(%)** ***N*** **= 97**	***P*** **value**	**Overall, N (%)** ***N*** **= 177**	**Experienced violence, N (%)** ***N*** **= 50**	***P*** **value**	**Overall, N (%)** ***N*** **= 408**	**Experienced violence, N(%)** ***N*** **= 119**	***P*** **value**
Aged < 18 years	223 (31.2%)	40 (41.3%)	**0.029**	40 (22.6%)	16 (32.0%)	**0.049**	63 (15.5%)	17 (14.3%)	0.865
Can read and write	693 (97.1%)	93 (95.9%)	0.700	172 (97.2%)	46 (92.0%)	**0.040**	397 (97.3%)	119 (100%)	0.065
Regular source of income	80 (11.2%)	10 (10.3%)	0.864	17 (9.6%)	7 (14.0%)	0.258	67 (16.4%)	14 (11.8%)	0.109
Consumed alcohol almost every day or every day in last month	2 (0.3%)	1 (1.0%)	0.253	11 (6.2%)	4 (8.0%)	0.508	121 (29.7%)	46 (38.7%)	**0.012**
Ever pregnant	189 (26.5%)	32 (33.0%)	0.137	70 (39.5%)	21 (42.0%)	0.734	234 (57.4%)	84 (70.6%)	**< 0.001**
Currently student	203 (28.4%)	27 (27.8%)	1.000	30 (16.9%)	7 (14%)	0.657	33 (8.1%)	11 (9.2%)	0.556
Venue based hotspot	585 (81.9%)	81 (83.5%)	0.777	136 (76.8%)	41 (82%)	0.332	348 (85.3%)	101 (84.9%)	0.879

*P*-values from Chi-squared test. Significant correlates (*p* < 0.05) shown in bold. These are descriptive statistics for association

#### Physical violence

Drinking alcohol almost every day or every day in the last 1 month was associated with higher lifetime and last year experience of physical violence across subgroups. Ever being pregnant was significantly associated with higher rates of physical violence for both lifetime and last year for YCS, and was also associated with experience of lifetime physical violence for YSW. For YTS being recruited from a venue-based hotspot was associated with higher lifetime rates of experiencing violence. Among YSW having a regular source of income was associated with lower rates of lifetime physical violence, while being a student was associated with higher rates of physical violence in the last year.

Ever being pregnant remained significantly associated in multivariable regression analysis for YCS with higher rates of physical violence for both lifetime and last year. For YTS and YSW, all variables significant in the Chi-squared test was also included in the corresponding multivariable regression model, while for YSW being aged < 18 years was also associated with higher risk of physical violence in the past year.

#### Sexual violence

Being aged < 18 years was associated with experience of lifetime sexual violence for YCS (*p* = 0.029) and YTS (*p* = 0.049), as well as for experience of sexual violence in the past year for YCS (*p* = 0.001). Literacy was associated with reduced experience of lifetime sexual violence for YTS (*p* = 0.040). For YTS, drinking alcohol almost every day or every day in the last 1 month was borderline significant for experience of sexual violence in the last year (*p* = 0.052) although the number of respondents was very small. For YSW, drinking alcohol almost every day or every day in the last 1 month was associated with higher rates of lifetime experience of sexual violence (*p* = 0.012), while ever being pregnant was associated with higher rates of both lifetime (*p* < 0.001) and last year experience of sexual violence (*p* = 0.031).

For each subgroup, the multivariable regression model for lifetime experience of sexual violence contained the dependent variables that were significant in the Chi-squared analysis. Being aged < 18 years and drinking alcohol almost every day or every day in the last 1 month were both associated with higher experience of sexual violence in the last year for YCS, while for YCS only drinking alcohol almost every day or every day in the last 1 month was associated in the final multivariable model with higher risk of sexual violence in the last year. For FSW ever being pregnant was the only variable that remained in the multivariable model for higher risk of sexual violence in the last year.

## Discussion

In this paper we have shown the prevalence of physical, sexual and police violence in different subgroups of AGYW who report ever having had sex in Mombasa, Kenya. Overall, high levels of physical, sexual and police violence were seen across AGYW engaging in casual sex, transactional sex and sex work. Despite their young age, almost a fifth of the respondents had experienced physical and sexual violence. Other studies from Kenya have also found high prevalences of experience of physical and sexual violence among AGYW [7]. Police violence (assault or arrest) was reported by one third of those questioned (this question was not administered with YCS respondents) in our study, similar to [21], where female sex workers in Kenya reported higher police violence compared to other forms of violence [21]. Overall, this study in particular highlights a high vulnerability to violence early on in life among all the subgroups of AGYW which needs to be addressed urgently through high quality programmes and policies.

For each kind of violence, and across subgroups, half or more of the respondents experiencing that form of violence experienced it within the last 1 year. This could be because of their young age, as their first experience of violence could have been within the last year. It is also concerning to note that overall a higher proportion of the respondents reported experiencing lifetime sexual violence than lifetime physical violence, although YSW reported equal lifetime levels of both forms of violence. This is different from [7] which found much higher physical violence among AGYW in Kenya than sexual violence, though this difference may be explained by differences in where participants were recruited, as in the previous study data was collected at home, whereas in this study it was collected at hotspots where people sought sexual partners or had sex. Also of concern, YCS and YTS aged < 18 years reported more sexual violence, which may be either due to increasing levels of violence among women aged < 18, or to older AGYW being more reluctant to report such episodes. Prevalence of violence was higher among YSW and YTS, consistent with findings from literature that selling sex and engaging in transactional sex can increase the risk of violence among AGYW [12, 22].

Our findings also show that for a high proportion of respondents, episodes of physical and sexual violence were accompanied by vaginal and/or anal sex. The combination of sex and violence makes the situation complex for girls and women and increases their vulnerability, potentially leading to coerced or forced sex with difficulties negotiating condom use [23–25]. Though reported condom use increased during the sexual intercourse at most recent physical and sexual violence in our study, half of the respondents still reported condomless sex. These findings are consistent with the existing literature, where violence is consistently associated with condom non-use [26] and condom breakage [27, 28]. Literature also shows that violence and condomless sex also increases women’s vulnerability to HIV, other sexually transmitted infections and negative reproductive health outcomes such as unwanted pregnancies, abortions and low birthweight babies [1, 8]. In our study, YSW and YTS more frequently reported vaginal or anal sex at first and most recent physical and sexual violence than YCS, suggesting that sex work and transactional sex increases girls’ and women’s vulnerability to violent sex. However, only a third of YTS and YCS reported using a condom during the most recent episode of sexual violence, substantially lower than reported by these groups in the absence of violence, and raising issues related to lack of negotiation power or skills. Overall, all groups experienced high rates of condomless sex during episodes of physical and sexual violence, increasing their risk of STIs, HIV, and other negative health outcomes.

The most common perpetrators of violence in our study were intimate partners (husband, boyfriend, spouse). In addition, regular non-client partners were identified as a main perpetrator of physical and sexual violence by YTS. YSW identified first-time paying client and regular paying clients as other key perpetrators of physical and sexual violence. Existing literature on violence against women and girls confirm that perpetrators are usually boyfriends, romantic partners, neighbours or family members [7, 24], while published works on violence against female sex workers identify clients and police as key perpetrators of violence [29] and show that consequences to violence differ by perpetrator [29, 30], although violence perpetrated by non-paying partners was more likely to be undisclosed than violence perpetrated by paying partners/strangers [31].

Consistent with findings from other studies, we found alcohol use to be associated with risk of physical and sexual violence over both lifetime and last year [32–36]. Daily use of alcohol may directly lead to violence, or young women may use alcohol as a coping mechanism for the acts of violence they have experienced. Studies have shown that women who have been subjected to gender-based violence often adopt risky behaviors such as alcohol abuse, which in turn can lead to more unprotected sex and an increased risk of acquiring HIV [37]. We also found that ever being pregnant was associated with higher rates of physical and sexual violence in some subgroups of respondents. Though literature has linked lack of control over sexual and reproductive choices and increased risk of unwanted pregnancies with exposure to violence [24, 35], a cross-sectional study like this can only show correlation and not causation. Having a regular source of income other than sex work was negatively associated with experience of lifetime physical violence among YSW. This is similar to other studies which have related main or sole dependence on sex work as an income source with high violence among female sex workers [38].

There are several limitations to this study. Firstly, the cross-sectional nature of the study precludes assessing causation between violence and associated factors. Face-to-face interviews were used, which may lead to overreporting of behaviours such as condom use due to social desirability bias. Participants were asked questions about their experiences of physical and sexual violence, which are traumatic events that participants may in some cases have been reluctant to answer. Although participants were young, and thus the time since first violence experiences was generally short, there may still have been recall bias in some cases. While the definitions of the YSW, YTS and YCS groups were based on existing literature [18, 19], this grouping of participants may also obscure a spectrum of behaviours: for example YSW may also engage in transactional sex, while women in the YCS group may find long-term partners. Another limitation is that we asked about violence by first and last perpetrator, which may underestimate the rate of IPV among YSW and YTS who may experience violence from multiple types of perpetrator. The questions on sexual and physical violence were single items and this may have underestimated the prevalence of violence and could be a limitation. Finally, while low income is an important factor increasing vulnerabilities, potentially including to physical and sexual violence, we only asked about regular income, and thus were unable to further explore this area. However, the study also has a number of strengths. It represents the first time that data has been collected at hotspots specifically on AGYW, while by collecting data on two separate occurrences of violence - the first and most recent – the study allows insight into how experiences of violence may have changed over time. Finally, a large sample of almost 1300 AGYW was collected, with substantial numbers of respondents in each subgroup including young sex workers who are an under-researched population, and thus the study is well positioned to provide important insights into these subgroups of AGYW.

Addressing and preventing different forms of violence in the lives of AGYW is crucial as violence is a human rights and public health concern. Both evidence from research and programmatic experience show that violence against women and girls can be prevented through interventions targeting the key drivers of violence in the setting [36, 38, 39]. Intimate partner violence, which was the main constituent of violence measured in our study, can be reduced by measures building girls’ and women’s agency, participation, and leadership; keeping girls in school and providing comprehensive sex education; and by scaling up and integrating HIV with sexual and reproductive health services for girls and women [39]. However, identifying and ensuring participation of AGYW at highest risk of violence and exploitation is important [29]. The high levels of exposure to violence seen in this study suggest that interventions with AGYW could also use geo-specific evidence to implement their interventions in hotspots. Combining interventions to reduce violence synergistically, informed by knowledge of where and how to find AGYW at risk of violence, has the potential to provide many public health and human rights benefits.

## Conclusions

Physical and sexual violence negatively impact AGYW and act as a barrier to their access to health, social entitlements and equal participation. Our study findings add to the body of evidence on violence against adolescent girls and young women, and provide new information on prevalence, perpetrators and predictors of physical and sexual violence across a varied sub-population within AGYW. Our study findings also add to the limited body of evidence on young sex workers, which is generally a very poorly researched population. There are successful models of interventions which have been designed to prevent and respond to violence against girls and women, and these can be adapted to this context. Empowering the girls and women with information and skills, creating economic opportunities, engaging the communities to address negative gender norms, providing supportive services and changing laws and policies to support women and girls can prevent violence against women and girls [38]. The findings of the present study are important to adapt and implement such interventions to prevent and respond to violence based on best practices.

## Supplementary information

**Additional file 1.** Table showing potential correlates of last one year experience of physical and sexual violence by subgroup.

## Data Availability

The datasets used and/or analysed during the current study are available from the corresponding author on reasonable request.

## Abbreviations

AGYW: Adolescent girls and young women
GBV: Gender-based violence
YCS: Young women engaged in casual sex
YTS: Young women engaged in transactional sex
YSW: Young women engaged in sex work (YSW)
