# Supplementary material for: Prevalence and patterns of gender-based violence across adolescent girls and young women in Mombasa, Kenya
Source: BMC Womens Health. 2020 Oct 12;20:229. doi: 10.1186/s12905-020-01081-8 (PMC7549220; doi:10.1186/s12905-020-01081-8)
Supplement: Supplementary file 1 — Additional file 1. Table showing potential correlates of last one year experience of physical and sexual violence by subgroup. [file 12905_2020_1081_MOESM1_ESM.docx]

# Appendix

Supplement Table 1: Potential correlates of last one year experience of physical and sexual violence by subgroup. P-values from Chi-squared test. Significant correlates (p<0.05) shown in bold.

| **Experienced physical violence in the last 1 year** | **YCS** | | | **YTS** | | | **YSW** | | |
| --- | --- | --- | --- | --- | --- | --- | --- | --- | --- |
|  | **Overall, N (%) N=714** | **Experienced violence,**  **N (%) N=39** | **P value** | **Overall, N (%) N=177** | **Experienced violence,**  **N (%) N=21** | **P value** | **Overall, N (%) N=408** | **Experienced violence,**  **N (%) N=79** | **P value** |
| Aged <18 years | 223 (31.2%) | 13 (33.3%) | 0.920 | 40 (22.6%) | 5 (20.0%) | 0.849 | 63 (15.4%) | 17 (21.5%) | 0.100 |
| Can read and write | 693 (97.1%) | 38 (97.4%) | 1.0 | 172 (97.2%) | 21 (100%) | 0.856 | 397 (97.3%) | 79 (100%) | 0.229 |
| Regular source of income | 80 (11.2%) | 4 (10.3%) | 1.0 | 17 (9.6%) | 3 (14.3%) | 0.431 | 67 (16.4%) | 10 (12.7%) | 0.398 |
| Consumed alcohol almost every day or every day in last month | 2 (0.3%) | 2 (5.1%) | **0.003** | 11 (6.2%) | 5 (23.8%) | **0.004** | 121 (29.7%) | 32 (40.5%) | **0.028** |
| Ever pregnant | 189 (26.5%) | 16 (41.0%) | **0.040** | 70 (39.5%) | 10 (47.6%) | 0.479 | 234 (57.4%) | 48 (60.8%) | 0.528 |
| Currently student | 203 (28.4%) | 7 (17.9%) | 0.148 | 30 (16.9%) | 2 (9.5%) | 0.536 | 33 (8.1%) | 11 (13.9%) | **0.041** |
| Venue based hotspot | 585 (81.9%) | 34 (87.2%) | 0.521 | 136 (76.8%) | 19 (90.5%) | 0.168 | 348 (85.3%) | 65 (82.3%) | 0.382 |
| **Experienced sexual violence in the last 1 year** | **YCS** | | | **YTS** | | | **YSW** | | |
|  | **Overall, N (%) N=714** | **Experienced violence,**  **N (%) N=44** | **P value** | **Overall, N (%) N=177** | **Experienced violence,**  **N (%) N=25** | **P value** | **Overall, N (%) N=408** | **Experienced violence,**  **N (%) N=58** | **P value** |
| Aged <18 years | 223 (31.2%) | 24 (54.5%) | **0.001** | 40 (22.6%) | 6 (24.0%) | 0.760 | 63 (15.4%) | 9 (15.5%) | 1.0 |
| Can read and write | 693 (97.1%) | 43 (97.7%) | 1.0 | 172 (97.2%) | 23 (92.0%) | 0.339 | 397 (97.3%) | 58 (100%) | 0.330 |
| Regular source of income | 80 (11.2%) | 3 (6.8%) | 0.462 | 17 (9.6%) | 5 (20.0%) | 0.070 | 67 (16.4%) | 8 (13.8%) | 0.703 |
| Consumed alcohol almost every day or every day in last month | 2 (0.3%) | 1 (2.3%) | 0.120 | 11 (6.2%) | 4 (16.0%) | 0.052 | 121 (29.7%) | 22 (37.9%) | 0.162 |
| Ever pregnant | 189 (26.5%) | 10 (22.7%) | 0.724 | 70 (39.5%) | 13 (52.0%) | 0.190 | 234 (57.4%) | 41 (70.7%) | **0.031** |
| Currently student | 203 (28.4%) | 16 (36.4%) | 0.231 | 30 (16.9%) | 4 (16%) | 1.0 | 33 (8.1%) | 6 (10.3%) | 0.444 |
| Venue based hotspot | 585 (81.9%) | 38 (86.4%) | 0.546 | 136 (76.8%) | 18 (72%) | 0.609 | 348 (85.3%) | 50 (86.2%) | 1.0 |

Supplement Table 2: Final multivariable regression model for each violence outcome for each population subgroup

| **Outcome variable** | **Dependent variable** | **Coefficient (95% CI)** | **P-value** |
| --- | --- | --- | --- |
| ***Ever experienced physical violence*** | *YCS* | | |
|  | - Ever pregnant | 2.106 (1.269-3.459) | 0.003 |
|  | *YTS* | | |
|  | - Consumed alcohol almost every day or every day in last month | 6.536 (1.832-26.646) | 0.005 |
|  | - Venue based hotspot | 2.921 (1.050-10.426) | 0.061 |
|  | *YSW* | | |
|  | - Regular source of income | 0.479 (0.238-0.905) | 0.030 |
|  | - Consumed alcohol almost every day or every day in last month | 2.080 (1.313-3.294) | 0.002 |
|  | - Ever pregnant | 2.019 (1.285-3.216) | 0.026 |
| ***Ever experienced sexual violence*** | YCS | | |
|  | - Aged <18 years | 1.670 (1.070-2.585) | 0.022 |
|  | YTS | | |
|  | - Aged <18 years | 2.565 (1.182-5.533) | 0.016 |
|  | - Can read and write | 0.074 (0.004-0.525) | 0.022 |
|  | *YSW* | | |
|  | - Consumed alcohol almost every day or every day in last month | 1.734 (1.091-2.748) | 0.019 |
|  | - Ever pregnant | 2.152 (1.366-3.442) | 0.001 |
| ***Experienced physical violence in the last 1 year*** | YCS | | |
|  | - Ever pregnant | 2.021 (1.026-3.892) | 0.037 |
|  | YTS | | |
|  | - Consumed alcohol almost every day or every day in last month | 8.750 (2.221-34.79) | 0.002 |
|  | YSW | | |
|  | - Aged <18 years | 1.958 (1.009-3.689) | 0.041 |
|  | - Consumed alcohol almost every day or every day in last month | 2.058 (1.211-3.481) | 0.007 |
|  | - Currently student | 2.278 (1.003-4.940) | 0.041 |
| ***Experienced sexual violence in the last 1 year*** | YCS | | |
|  | - Aged <18 years | 2.815 (1.517-5.276) | 0.001 |
|  | - Consumed alcohol almost every day or every day in last month | 14.0419347 (0.516-382.2) | 0.072 |
|  | YTS | | |
|  | - Consumed alcohol almost every day or every day in last month | 4.317 (1.033-16.43) | 0.033 |
|  | YSW | | |
|  | - Ever pregnant | 1.977 (1.097-3.704) | 0.027 |
